# Pathway Analysis of Genes Identified through Post-GWAS to Underpin Prostate Cancer Aetiology

**DOI:** 10.3390/genes11050526

**Published:** 2020-05-08

**Authors:** Samaneh Farashi, Thomas Kryza, Jyotsna Batra

**Affiliations:** 1 School of Biomedical Sciences and Institute of Health and Biomedical Innovation, Queensland University of Technology, Brisbane, Queensland 4059, Australia; samaneh.farashi@hdr.qut.edu.au (S.F.); thomas.kryza@mater.uq.edu.au (T.K.); 2 Translational Research Institute, 37 Kent Street, Woolloongabba, Queensland 4102, Australia; 3 Mater Research Institute, University of Queensland, Translational Research Institute, 37 Kent Street, Woolloongabba, Queensland 4102, Australia

**Keywords:** prostate cancer, post-GWAS, functional variants, pathway analysis, upstream analysis, Oncomine

## Abstract

Understanding the functional role of risk regions identified by genome-wide association studies (GWAS) has made considerable recent progress and is referred to as the post-GWAS era. Annotation of functional variants to the genes, including *cis* or *trans* and understanding their biological pathway/gene network enrichments, is expected to give rich dividends by elucidating the mechanisms underlying prostate cancer. To this aim, we compiled and analysed currently available post-GWAS data that is validated through further studies in prostate cancer, to investigate molecular biological pathways enriched for assigned functional genes. In total, about 100 canonical pathways were significantly, at false discovery rate (FDR) < 0.05), enriched in assigned genes using different algorithms. The results have highlighted some well-known cancer signalling pathways, antigen presentation processes and enrichment in cell growth and development gene networks, suggesting risk loci may exert their functional effect on prostate cancer by acting through multiple gene sets and pathways. Additional upstream analysis of the involved genes identified critical transcription factors such as HDAC1 and STAT5A. We also investigated the common genes between post-GWAS and three well-annotated gene expression datasets to endeavour to uncover the main genes involved in prostate cancer development/progression. Post-GWAS generated knowledge of gene networks and pathways, although continuously evolving, if analysed further and targeted appropriately, will have an important impact on clinical management of the disease.

## 1. Introduction

Prostate cancer (PrCa) is the second leading cause of cancer death among men in the western world [1]. Genetic and non-genetic (environmental) risk factors are known to be involved in PrCa, with the prominent effect of genetics demonstrated by 57% heritability that has been discovered by the large-scale twin studies evaluating the role of genetics in PrCa development [2,3]. To discover genetic factors, during the last decade, genome-wide association studies (GWAS) have successfully identified >160 loci associated with the risk of PrCa [4].

A locus of multiple single nucleotide polymorphisms (SNPs) within a linkage disequilibrium (LD) block is represented by a so-called tag SNP that pinpoints the associated risk region [5]. By imputation analysis, the SNPs in LD are included in the association analyses. One of the difficulties associated with LD patterns is to identify the exact functional variant of a GWAS–SNP signal, particularly for variants in strong LD with biologically causal variants. In addition, the LD patterns throughout the genome reflect the population history and therefore differ in population sub-groups. Fine-mapping studies assist post-GWAS characterisation of the genes/proteins, which are influenced by a particular SNP incorporating the LD pattern of population sub-groups, in further post-GWAS analyses. Another ongoing challenge of post-GWAS is the identification of the target genes for the SNPs located within the non-coding regions of the genome. The functional variants are mainly located within non-coding regions [6,7] with the prominent consequences due to (i) change in transcription factors (TFs) binding site [7,8], (ii) change in DNA methylation marks [9], and (iii) chromatin architecture alteration [10] or a combination of above-mentioned mechanisms. 

The non-coding functional variants are mainly involved in regulating gene expression by changing chromatin interactions and/or altering the binding of certain TFs that consequently lead to modulation of the expression of the target genes. Therefore, many post-GWAS studies include the expression quantitative trait loci (eQTLs) as functional variants, representing the regulatory impact of germline loci associated with gene expression levels. The eQTLs can affect the target genes directly (cis-eQTLs) and indirectly (trans-eQTLs). For example, SNP rs55958994 is a PrCa-risk locus, which affects the expression of several genes such as *CNTN1*, *KRT8*, *FAIM2*, *KRT7*, *ITGA5* and *KRT18* that are located on the same chromosome [11]. In addition, this SNP is involved in regulating two genes (CDH23 and SIPA1) on different chromosomes via long-range chromatin interactions (i.e., trans-eQTLs) [11].

More recently, the transcriptome-wide association studies (TWAS) approach has been used [12,13] to investigate the association of gene expression with PrCa-risk to discover independent genes from a previously reported risk variant [4]. While current techniques can help to refine the role of PrCa–GWAS loci in prostate tumorigenesis, there is still a majority of unknown genes, in particular, non-coding RNAs (ncRNAs) in the vicinity or within the distance of the risk loci, yet to be discovered [14]. This brings up the urgent need for other approaches implementing the GWAS and post-GWAS data to improve the clinical management of PrCa. In particular, pathway-based analysis of GWAS assigned genes has been used to define a group of genes that are involved in the same biological and/or molecular processes in prostate tumorigenesis [15,16]. Notably, mapping GWAS genes into gene networks [17] and molecular pathways [18] can increase the understanding of risk loci in PrCa biology. 

GWAS have been successful in revealing new treatment targets in PrCa [4]. To a higher level, utilising post-GWAS data that is the biologically active part of the risk regions can provide us with undeniable benefits in drug repurposing to reveal putative targets. Furthermore, investigating the biological pathways that post-GWAS genes act through can uncover future successful drug targets. As an example, functional variants affecting oncogene *MYC* [19] or androgen receptor (*AR*) [20] may contribute to the role of these genes in the related pathways in PrCa. In addition, post-GWAS have identified functional variants within genes encoding PrCa biomarkers such as MSMB and KLK3. The encoded proteins by these genes are known to be involved in cancer cell proliferation, invasion or metastasis, and, therefore, modifications exerted by the functional variants that change the produced proteins’ function may be explained by post-GWAS [14,21,22]. 

These examples suggest that investigation of the pathways enriched in such proteins in PrCa can significantly increase the chances for clinical success and productivity for this highly polygenic cancer [23]. Currently, there are several algorithms to investigate the pathway and gene network enrichments in a group of genes. We used the Ingenuity Pathway Analysis (IPA) [24] and Gene Set Enrichment Analysis (GSEA) [25] algorithms, which are amongst the most commonly used tools. We further utilised the Oncomine dataset to investigate the expression status of post-GWAS assigned genes studied here in prostate primary and metastatic clinical samples [26]. The findings may provide us with clues of new pathways together with our current knowledge of well-established pathways involved in prostate tumour development, represented by several crucial genes affected by functional risk variants.

## 2. Materials and Methods

### 2.1. Prostate Cancer Risk Associated, Functional SNPs and Genes

Available published post-GWAS data in PrCa was integrated to compile a list of functional SNPs that have been assigned until October 2019. These genes were identified SNPs and have been assigned to the related gene (described as post-GWAS genes in this study) until October 2019 (Table S1). The period of identification of these genes, from 2003 to 2019 by 35 studies, is listed in Table S1. We have included the post-GWAS data carried out by meta-analysis of previous GWAS, which conducted the resequencing or fine-mapping of the risk-loci to identify independent PrCa-associated variants [13,27]. The GWAS data in PrCa represents mainly the European population structure; therefore, we restricted our focus to the post-GWAS studies that have been conducted in European ethnicity. This is reflected in imputation and fine-mapping analyses conducted in European ancestry. The majority of these variants have been found by eQTL analysis of the risk loci that contribute to the expression of the first category of genes studied here. The second category of genes is those assigned to functional variants identified by TWAS [12,13]. The third category consists of 70 genes that are assigned to 68 individual risk loci by different experimental strategies detailed in Table S1. The fourth and minority category of genes were identified by in-silico annotation strategies. These functional variants are located within protein-coding regions (synonymous or non-synonymous nucleotide changes) that may affect the structure, biochemical properties (e.g., charge) or the stability of the produced protein of a given gene and subsequently modify the molecular function of the protein [21]. This fourth group in the study consists of nine genes/proteins affected by nine SNPs. Some of the post-GWAS assigned genes have been reported in separate studies using different approaches and, therefore, may belong to multiple categories of the genes defined in this study. 

### 2.2. Pathway Analysis

The post-GWAS pathway analysis approach was applied to the post-GWAS assigned genes. We used IPA and GSEA, described below, to explore the relevant pathway/gene network enrichments of these genes at FDR <0.05 throughout the analyses. The *p*-values represent the probability for each result and are corrected for the multiple testing (Benjamini–Hochberg method) that arises from evaluating the submitted list of genes against every pathway (Tables S2–S5). The implicated canonical pathways and gene networks from the post-GWAS assigned genes were analysed, including and excluding major histocompatibility complex (*HLA*) genes. The latter analysis was performed to avoid the possible effect of the relatively high number of *HLA* genes (14 *HLA*s) on the results.

Ingenuity Pathway Analysis (IPA) was used to measure the statistical significance of the relationship pattern of the proteins produced by the genes studied here and matched with the prior published data [24]. IPA is based on experimentally validated pathway enrichments that include an upstream regulatory analysis of the genes. In addition, we investigated the upstream regulators of the genes in the context of the related pathways. IPA scores the upstream regulators, based on their statistical significance, by measuring the overlap of observed and predicted regulated gene sets, as previously described [24]. We considered direct and indirect relationships (expanding predictions to include intermediate molecule(s) based on experimental data) that have been observed and experimentally validated.

Gene Set Enrichment Analysis (GSEA) was used to determine whether a prior defined set of genes and our gene list of interest displayed statistically significant, concordant alterations in gene expression associated with a disease that manifests at the level of biological pathways or co-regulated gene sets [28]. GSEA includes the Kyoto Encyclopedia of Genes and Genomes (KEGG) [29], Gene Ontology (GO) [30] and REACTOME [31] datasets in the analysis. Gene set enrichment in GSEA analysis uses prior gene sets that have been grouped together by their involvement in the same biological pathway or by proximal location on a chromosome [25]. 

GSEA converted the submitted genes into Entrez genes (Table S1) for further annotation analysis of their significance in a pathway/network based on the Molecular Signatures Database (MSigDB) [32]. In addition, the following analyses available in GSEA were carried out for post-GWAS identified genes: “hallmark gene sets” and “computational gene set” analysis, which considers cancer modules, annotated to the oncogenic signatures. GSEA evaluates the overlap of the provided genes with a given known pathway/gene network and estimates the statistical significance at FDR < 0.05. The GO analysis was used to investigate the function of genes (corresponding proteins) in *homo sapiens*, their ontology and the involved pathways [30]. The enrichments of post-GWAS genes in the GO biological process, GO cellular component and molecular function were investigated. 

### 2.3. Expression Analysis of the Post-GWAS Identified Genes in Clinical Samples

We utilised the Oncomine microarray database (http://www.oncomine.org) in PrCa to investigate the expression of the post-GWAS genes in patient samples. The Oncomine datasets are derived from differential expression analyses that compared defined samples in groups of cancerous, normal and metastatic tissues or cell lines [26]. The Oncomine originated datasets used in the study are (1) the Taylor dataset (Oncomine ID: n9205), including 150 prostate carcinoma tissue specimens (131 specimens from primary and 19 metastatic tumours) and 29 paired normal adjacent prostate tissue specimens [33]; (2) the Yu dataset (Oncomine ID: n5345) that used 23 normal prostate and 64 prostate carcinoma samples [26]; (3) the Grasso dataset (Oncomine ID: n6252) that describes 59 localised prostate carcinoma and 28 benign prostate tissue specimens [34]. We used three studies with the highest number of patient samples to investigate the dysregulated genes that are also overlapped with post-GWAS genes. Moreover, these studies provide samples from primary tumours, normal and metastatic tissues in patients. The over and under-expressed genes in each study were filtered for significant differentially expressed genes in primary tumours versus normal/metastatic samples (*p*-value < 0.05) by more than 1.5 fold change. The comparison of post-GWAS dysregulated genes was performed separately for primary tumours versus normal or metastatic samples investigating common genes based on Entrez gene identifiers. 

## 3. Results

**Figure 1:**
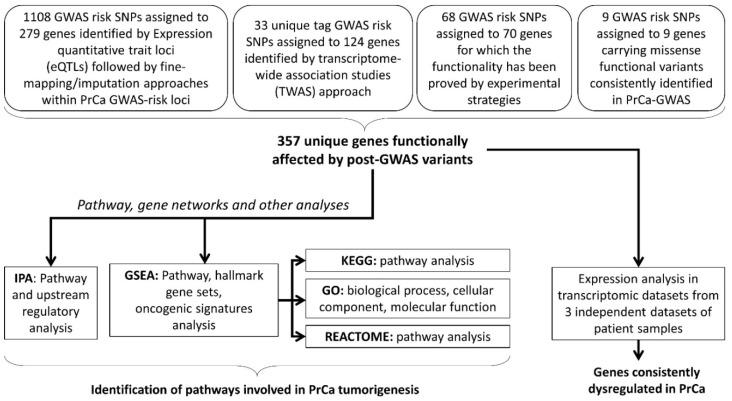
The study design. This flow chart depicts the flow of the analyses that were performed in this study. Assigned genes represent the functional variants that contribute to prostate cancer (PrCa) tumorigenesis via (i) regulating the target genes through the expression quantitative trait loci (eQTLs), (ii) transcriptome-wide association study (TWAS) of the PrCa-risk loci, or (iii) a functional impact evaluated by experiments, or (iv) in-silico studies. Further pathway analysis was performed using different algorithms. In addition, investigating the expression status of the assigned genes demonstrated several overlapped dysregulated genes between three expression datasets and post-GWAS genes. Note: some of the genes have been reported in studies utilising multiple approaches, thus belong to multiple categories that are included in this study. GWAS: genome-wide association studies, IPA: Ingenuity Pathway Analysis, GSEA: Gene Set Enrichment Analysis, KEGG: Kyoto Encyclopedia of Genes and Genomes, GO: Gene Ontology.

We undertook a post-GWAS pathway analysis approach, including all the genes assigned to functional variants discovered so far, to the best of our knowledge (Figure 1) [12,14]. These genes, included in this study, belong to four different categories based on the strategies they have been discovered previously. In total, 357 genes assigned to PrCa-functional risk variants are compiled in this study to provide a testable hypothesis for pathway analysis and future investigations on PrCa aetiology. The first category is GWAS–eQTL pairs as functional variants that are likely to affect PrCa-risk through their effects on gene expression. The GWAS–eQTL data has been identified based on information on eQTLs and generated by extended analysis (i.e., imputation and fine-mapping methods for the European population) on original GWAS data [27,35,36]. These eQTLs consist of the majority of the functional variants studied here, which is 1108 SNPs assigned to 279 individual genes. The second category is the reported risk variants in PrCa that have been further integrated with expression data (i.e., TWAS). Out of 357 genes, 124 individual genes, assigned to 33 individual SNPs, have been identified by TWAS [12,13]. In the third category, 68 functional GWAS-identified variants, assigned to 70 individual genes, have been discovered by further experimental validation such as chromatin immunoprecipitation (ChIP) sequencing. In the fourth category, there are 9 GWAS risk loci that generate missense nucleotide changes in 9 key proteins in prostate tumorigenesis (Figure 1) [12,14]. Among the functional SNPs in the first category, GWAS–eQTL pairs, there are 6, 3 and no functional SNPs overlapped with the second, third and fourth categories, respectively. Including these genes, we aimed to identify the possible pathways that are enriched for the PrCa-risk in addition to dysregulated genes and, therefore, likely contributing to the development of PrCa (Table S1). 

### 3.1. Pathways and Gene Set Enrichments Including HLA Genes

**Figure 2:**
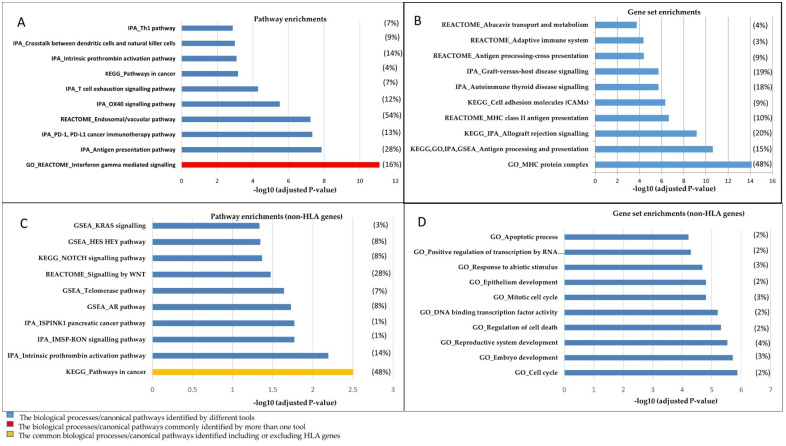
The top 10 significant canonical pathways and gene sets resulting from algorithms used in this study. The diagrams illustrate (**A**) enrichments in pathways and (**B**) gene sets in biological processes, including HLA genes. (**C**,**D**) represent the analyses conducted while excluding HLA genes. The significant pathways/gene sets have been depicted in the graphs based on –log10 (*p*-value). A full list of the pathways and gene networks has been represented in Tables S2 and S3. The algorithm that has been used for each pathway/gene set is shown as a prefix. The ratios of post-GWAS/total genes involved in a pathway/gene set are presented in parentheses for a given pathway/gene set. The average of this ratio is presented for a pathway/gene set resulted from more than one tool. Note: To avoid over-presentation of the GO results, we depicted all immune system-related gene sets resulting from GO as antigen processing and the presentation gene set.

IPA mapped 357 genes into 336 Entrez gene identifiers to the known pathways; 21 genes were not included in IPA analysis, with the majority of long ncRNAs as unmapped genes (Table S1). Pathways related to immune signalling, such as interferon-gamma (INFG) mediated signalling, antigen processing and presentation pathways, were identified as the top-ranked significant pathways (Figure 2A, Table 1). GSEA identified 354 genes out of 357 submitted genes and converted them into 332 NCBI/Entrez gene identifiers; 22 genes were not included by GSEA. These were 22 long ncRNAs and 18 were common in both IPA and GSEA tools (Table S1). GSEA analysis revealed the androgen response as the most significant pathway (Table S3). The IFNG was the most significant pathway resulting from the REACTOME, KEGG and GO analyses and the fourth significant pathway in the GSEA analysis. Moreover, in both IPA and GSEA analyses, significant enrichment of immune response and cancer-related pathways/gene networks was demonstrated (Supplementary Data S2–S3). The results of both IPA and GSEA pinpoint several well-known pathways involved in PrCa as well as enrichments in cancer-related gene networks and upstream regulators (Tables S4 and S5). Of those, cancer immune system related programmed cell death protein 1 (PD-1) and its ligand (PD-L1), endosomal/vacuolar and OX40 signalling pathways were among the highly significant pathway enrichments (Figure 2A, Table S2).

The gene set analysis by GO presented the MHC protein complex as the most significant gene set (FDR: 7.48 × 10^−15^; Figure 2B). The antigen processing and presentation gene set were shown as a commonly identified gene set using all tools (Table 1). In addition, several observed gene sets involved in allograft rejection and cell adhesion molecules were identified as of high significance (Figure 2B). 

### 3.2. Pathway and Gene Set Enrichments of Non-HLA Genes

We excluded 14 *HLA* genes while performing pathway analysis to identify HLA independent key networks/pathways enriched in the post-GWAS assigned genes (non-HLA genes identified by post-GWAS are listed in Table S1). 

The results for non-HLA genes identified additional less-known pathways in PrCa, such as intrinsic prothrombin activation and telomerase pathways (Figure 2C), that are interesting subjects for further follow-up studies. The intrinsic prothrombin activation pathway demonstrated as the most significant canonical pathway (FDR = 4.31 × 10^−6^) by IPA, is enriched in crucial proteins in PrCa, such as PIK3C2B, KLK3, RALB, NKX3-1, FGFR2, CREB3L4, CDKN1B, MAP2K1 and ATM (Tables S2 and S6). Additionally, the androgen-signalling pathway (AR pathway, Figure 2C) that is known to play a key role in PrCa [36,37] was identified as a highly significant pathway. Pathways in cancer were demonstrated as the top-ranked canonical pathway, analysing both the non-HLA genes and including HLA genes by KEGG.

Excluding HLA genes results in several gene sets involved in molecular mechanisms of cell death, development and mitotic cell cycle that were observed in this analysis (Figure 2D, Table S6). Additionally, the results from the gene set analysis revealed significant enrichments in components of the presentation and processing antigens via the estrogen receptor (ER) pathway and allograft rejection gene sets. 

### 3.3. Gene Network and Upstream Regulatory Analysis

**Figure 3:**
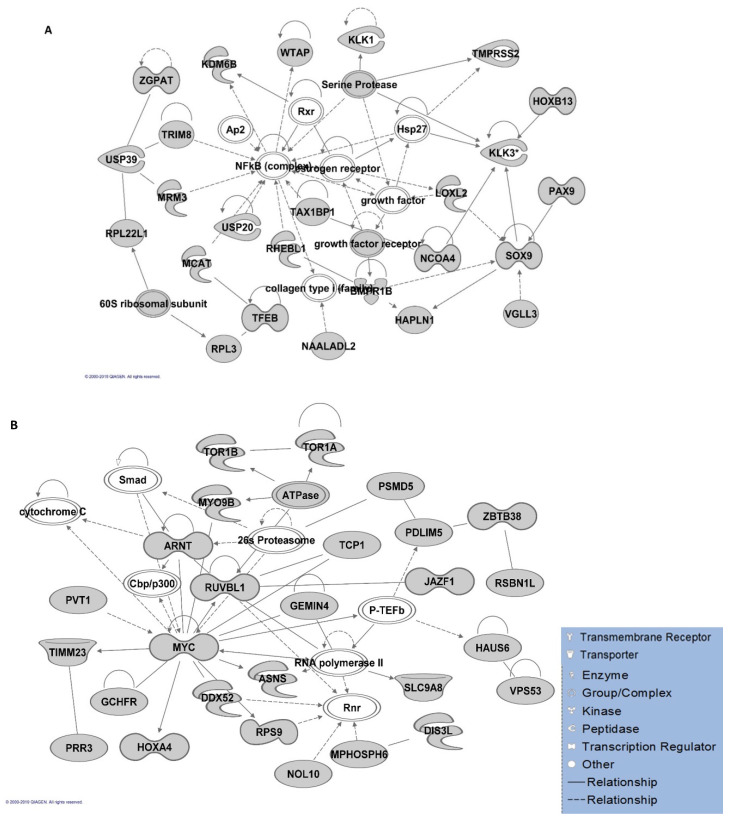
Ingenuity Pathway Analysis (IPA) gene network analysis. A map of the top-ranked gene network in IPA analysis with the highest number of the involved genes (**A**) including major histocompatibility complex (HLA) genes and (**B**) non-HLA genes. Arrows depict protein–protein interactions of molecules (in grey) produced by the post-GWAS assigned genes. Solid and dashed arrows between nodes represent direct and indirect interactions between molecules, respectively. The arrowheads depict an “act on” relationship towards positive regulations. The blind-ended arrows represent the inhibitory interactions. Bidirectional arrowheads indicate reversible reactions. The interactions are compact representations of literature-based knowledge. Each node represents a protein complex (illustrated in white).

Gene networks involved in different molecular and cellular functions, including connective tissue development and function and organ morphology, were identified by the IPA algorithm. However, cell morphology and cellular assembly/organisation were the most significant gene networks for non-HLA genes (Table S4). In addition, lipid metabolism, molecular transport and small molecule biochemistry were shown as the second top network for both analyses, including and excluding HLA genes. The interactions of the proteins involved in the top-ranked gene networks have been illustrated in Figure 3A,B.

**Figure 4:**
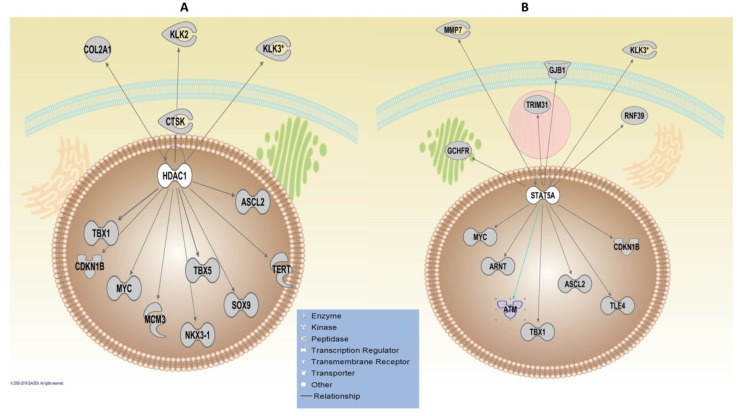
Ingenuity Pathway Analysis (IPA) upstream regulatory analysis. Upstream analysis of the post-GWAS genes, including HLA and non-HLA genes, demonstrated (**A**) HDAC1 and (**B**) STAT5A as the most significant transcription factors (TFs) that regulate the highest number (13 molecules illustrated above) of the post-GWAS genes studied here. The sub-cellular localisation of the molecules has been illustrated by pinpointing the broad network communication of the involved molecules in a cell. Arrows have depicted protein-protein interactions of molecules produced by the post-GWAS assigned genes. The arrowheads depict an “act on” relationship towards a positive regulation.

The upstream regulatory analysis using the IPA algorithm revealed WDR5 as the most significant TF, which was common in both analyses, including/excluding HLA genes with the FDR value of 0.01. WDR5 regulates five key genes, including IGF2R, KLK2, KLK3, MYC and TMPRSS2. Two TFs, activator of transcription 5A (STAT5A) and Histone deacetylase 1 (HDAC1), were revealed as significant TFs, regulating the highest number of the genes that are 13 genes in both analyses (Figure 4). The CTNNB1 was shown to regulate the highest number of genes (23 genes) (Figure S1); however, it was only significant when including HLA genes (FDR = 0.03). The adjusted *p*-value for CTNNB1 revealed a borderline significance threshold (FDR = 0.05) for the upstream regulatory analysis of non-HLA genes (Table S4). 

In GSEA analysis, TFs are demonstrated as the majority of proteins (gene family) encoded by the post-GWAS genes involved in related pathways (Table S7). In this analysis, “gene family” demonstrates a group of encoded genes that share a common feature, such as homology or biochemical activity. The AR pathway was a common pathway in HLA inclusive (FDR = 0.01) and non-inclusive (FDR = 0.02) analysis by GSEA. In addition, the AR was identified as an upstream regulator only in the full list of genes; however, it was not significant after multiple testing corrections. 

### 3.4. Expression Signature of the Post-GWAS Identified Genes in the Patient Samples

**Figure 5:**
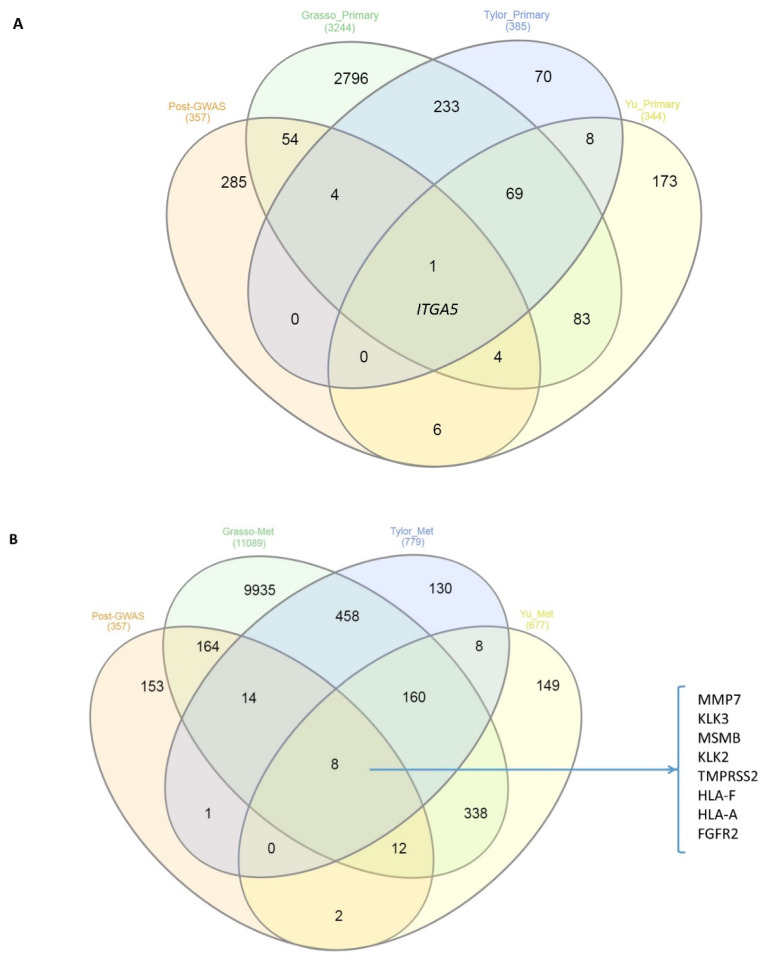
Venn diagram of the overlaps between post-GWAS assigned genes that have been studied here and the three Taylor, Grasso and Yu prostate datasets in (**A**) primary tumours vs. normal samples and (**B**) primary tumours vs. metastatic samples. The numbers in parentheses represent the numbers of significant (*p*-value < 0.05 by more than 1.5 fold change) differentially expressed genes. There is one gene (*ITGA5*) overlapping in the first comparison, while eight genes listed in (**B**) are up-or down-expressed commonly in metastatic samples of all three studies and post-GWAS assigned genes.

To evaluate whether the post-GWAS genes are differentially regulated genes in clinical samples, we used the Oncomine web-based dataset to investigate the possible overlaps between post-GWAS assigned genes and dysregulated genes in primary prostate tumours vs. normal or metastatic patient samples [26]. The comparison of differential gene expression of primary tumours vs. normal samples in Grasso et al. [34], Taylor et al. [33] and Yu et al. [26] identified 63, 5 and 11 genes, respectively, in common with post-GWAS genes. A similar investigation for primary vs. metastatic tumours in the above-mentioned three studies revealed 198 common genes for Grasso, 23 genes for Tylor and 22 genes for the Yu study. There was one overlapping gene (*ITGA5*) when we compared the post-GWAS genes and dysregulated genes in primary tumours vs. adjacent normal tissue resulting from the three studies, of Taylor, Grasso and Yu (Figure 5A). The same investigation for the post-GWAS vs. dysregulated genes in metastatic samples identified eight genes in common (Figure 5B). The identified genes are among well-known genes in PrCa or targets for therapeutic approaches, such as prostate-specific antigen (PSA, encoded by *KLK3*) inhibitors [26]. 

## 4. Discussion

In this study, we investigated networks and pathways, which resulted from post-GWAS assigned genes to demonstrate the biological and clinical relevance of functional variants in PrCa. With increasing success in cancer genomic data interpretation [14] and exponential improvements in post-GWAS studies [38], applying post-GWAS pathway analysis may help to reveal the full spectrum of germline variants’ role in prostate tumorigenesis. Our analyses highlighted the involvement of several known pathways in PrCa and pinpointed other less well-known pathways that might be valuable for researchers to explore new therapeutic targets in PrCa. Although while including HLA genes, some vital cancer-related signal transduction pathways such as Erk1/Erk2 MAPK and Notch signalling pathways were identified, the majority of the pathways and biological processes were involved in the immune response. While analysing the non-HLA genes, no enrichments in immune system-related pathways were shown in comparison to the gene list, including HLA genes (Tables S4 and S5). Notably, unlike the analysis including HLA genes, excluding HLA genes presented a higher number of cancer-related gene networks and pathways. Additionally, the cancer-related pathways that were identified by GSEA show a lower significance in comparison to immune system-related pathways. This difference may be explained by the possible effect of the relatively considerable number of HLA genes (14, consisting of 4% of the total number of the genes) on the results. For example, KRAS, WNT and Notch signalling pathways are well-established pathways in PrCa contributing to metastasis [39,40,41]. There is an urgent need to identify the mechanisms that promote angiogenesis and cell proliferation during PrCa metastasis from the primary tumour to the bone, which is the principal site of PrCa metastasis. Thus, further investigations on the findings from this study, focused on experimental strategies, might assist in addressing this need. 

The results pinpointed the AR pathway and AR as one of the upstream regulators. AR can modulate the expression of TFs, biomarkers and vital tumour promoters in PrCa development, such as KLK2, KLK3, MYC, MSMB and TMPRSS2 [42]. It is known that AR can activate other signalling cascades like the MAPK, Akt, JAK-STAT3 pathways [43,44] and stimulate growth processes in cells. This is suggestive of the role of functional variants in modulating the genes regulated by AR or implicated in the AR axis, which can be incorporated into currently used biomarkers stratifying metastatic PrCa patients [19]. 

In this study, the significant biological processes of the non-HLA genes are mainly recognised by GO. The GO analysis of a recent TWAS in PrCa by Mancuso N. et al. [12] demonstrated positive regulation of chromatin binding, nuclear membrane organisation and chaperone-mediated protein folding as top-ranked biological processes [12]. These processes were identified in our GO analysis when we excluded HLA genes; however, other gene sets such as cell cycle, regulation of cell death, embryo development and reproductive system development were the top-ranked gene ontologies (Supplementary Data S3–S4). Highly significant enrichments of antigen processing and presentation gene sets resulted from this study may suggest that focusing on post-GWAS data could reveal additional biological mechanisms related to immune response. Interestingly, PD-1 and the related PD-L1 cancer immunotherapy pathway was demonstrated as the second most significant pathway in IPA analysis. Similarly, the previous study by Schumacher F. et al. using GWAS risk loci, including some functional variants, identified the PD-1 signalling as the most significant pathway [45] in addition to the antigen processing, presentation and IFNG mediated signalling pathways [46]. Of the less well-known identified pathways resulting from this study, the OX40 signalling pathway that enhances the survival of T-cells in PrCa [44] is an interesting finding to add to the significant involvement of post-GWAS genes in the immune response. Remarkably, Phases 1 and 2 of clinical trials are targeting the OX40 signalling pathway in PrCa combination therapies [5]. The OX40 signalling pathway involves the binding of OX40L to OX40 receptors on T-cells, preventing them from dying and subsequently increasing cytokine production. Given the fact that a growing number of trials are ongoing with the immune checkpoint antibodies in PrCa, further exploration of immune system-related discovered pathways in this study can help greatly accelerate our ability to translate the genetic basis of PrCa to the clinic. 

The identification of critical TFs in this study implies the central importance of the upstream investigation of functional PrCa-risk loci. STAT5A, HDAC1 and Cyclin D1 (CCND1) were the common significant TFs revealed by IPA analysis, including HLAs and non-HLA genes. These TFs regulate high numbers of post-GWAS genes, including some vital genes in PrCa, such as *ATM*, *CDKN1B* and *ARNT*. STAT5A/B plays a critical role in prostate cell survival and tumour growth [46]. The therapeutic potential of STAT in cancer is under investigation in several clinical trials using STAT inhibitors [47]. HDAC1 exerts an androgen-dependent regulatory effect on prostate cell proliferation and development; thus, HDAC1 inhibitors have been suggested as a new therapeutic approach to study and further develop [48]. Interestingly, HDAC inhibitors have entered Phase-2 clinical trials as a new antineoplastic drug in PrCa treatment [4]. Moreover, the high activity of HDACs has been reported to cause epigenetic alterations associated with malignant PrCa cell behaviour [49]. Additionally, a high rate of HDAC1 expression has been significantly associated with tumour dedifferentiation [38]. Other TFs such as WDR5, TDP2, EED, SMARCD1 and NLRC5 that regulate well-known genes in PrCa, including *KLK3*, *TMPRSS2*, *MYC*, *MMP7*, are identified in upstream analysis using IPA. Given the fact that TF binding is key to gene expression reprogramming [47], disrupting crucial TFs might be the relevance of the regulatory role of PrCa post-GWAS loci [50]. The role of some of these proteins has been well-studied in PrCa [42]. For instance, NLRC5 is known to influence cytokine response via immune pathways and is suggested as a novel biomarker for cancer patient prognosis and survival [51]. In this way, assigned genes to post-GWAS loci may contribute to molecular and cellular biological processes leading to the overall outcome of prostate cell growth. In fact, the cumulative impact of post-GWAS genes on several main pathways, in addition to their upstream regulators, may influence prostate tumorigenesis/progression and will be a valuable avenue to explore combination therapies in the future [52]. For example, the IFNG-mediated signalling pathway relies on other signalling proteins such as JAK1, JAK2 and STAT-1 to induce the signal transduction pathways. Notably, the above-mentioned pathways are identified here, highlighting the importance of integrating the results to advance the current understanding of PrCa pathogenesis in order to improve the treatment strategies.

Accordingly, we believe that pathway analysis using post-GWAS data is an efficient approach for several reasons. First and most important, post-GWAS analysis enables us to focus on functionally involved genes in PrCa-risk and, therefore, will greatly speed up our ability to link the functional part of the genome into the clinic. Second, the reproducibility of GWAS is a valuable advantage, emphasising the high potential to reveal new discoveries from GWAS data. Third, dysregulation of PrCa-driving proteins, such as MMP7, MSMB and KLK3 [12,14] shown in our investigation using the Oncomine dataset, pinpoints the likely promising post-GWAS approach to highlight already identified gene networks and pathways for further follow-up in the clinic. However, the interpretation of the pathway-based analyses largely depends on the existing algorithms, which use various criteria to assign a gene to a network/pathway. The observed differences in pathway analyses, represented by different algorithms, may vary based on the specific methodologies. In particular, the main challenge in this type of analysis is that the observed outcomes depend on the input data (number of genes) recognised by different tools. In particular, ncRNAs are not recognised by IPA and GSEA. Thus, this caveat vastly deters our understanding of the role of ncRNAs identified by post-GWAS in the related pathways/gene sets, despite their critical effects on prostate tumorigenesis [53,54,55,56]. Available methods mostly have been developed for integrating protein-coding genes overlooking the contribution of ncRNAs to molecular pathways; therefore, there is an urgent need to include these molecules in pathway analysis methods. Moreover, the current approaches assign the functional SNPs to the nearby genes that overlook the active nature of the genome regardless of distance. Additionally, post-GWAS analysis suffers from the lack of data from non-European populations investigated in GWAS studies, limiting our ability to discover variants that may be relevant to PrCa in multi-ethnic populations. The research community has started to overcome this limitation by conducting GWAS in non-European populations [57,58,59,60,61]. 

The present study focuses on the genetics of PrCa that restrict the results to contribute to our current knowledge by only genetically related pathways. As we develop better strategies in post-GWAS, many more genes and their interactions with the non-genetic or environmental factors will be revealed, and we will get closer to discover a full spectrum of their mechanisms of action. Nevertheless, further investigations on findings in this study may help to link the genetic basis of PrCa into the molecular and cellular mechanisms in PrCa through related pathways. With the urgent need for personalised care for PrCa patients, additional screening and treatment approaches can strikingly modify the diagnosis protocol for a better estimation of disease progression [62]. Known pathways, in addition to as-yet-unknown pathways, may be leveraged to clarify the implication of various gene sets in PrCa and provide an idea for clinical development of pathway inhibitors [17]. For example, modifying HLA antigens, which demonstrate frequent alteration in PrCa patients [16], have been suggested to improve the efficacy of immune responses against PrCa [63]. Notably, in this study, the antigen presentation and immune response pathways were shown to be significantly enriched in post-GWAS risk loci of PrCa. New treatment strategies could be developed for subsets of these identified pathways that are yet to be tested in improving risk stratification.

## 5. Conclusions

Collectively, the results presented here suggest that post-GWAS pathway analysis may help to prioritise the critical networks of genes involved in relevant pathways for further translational studies. However, deeper investigations of the post-GWAS identified pathways in tumorigenesis are required to examine and validate their contribution. 

## Figures and Tables

**Table 1:** The top-ranked (the most significant) canonical pathways, gene sets and molecular functions that the post-GWAS assigned genes (described in Supplementary Table S1) are enriched in.

Tool	Top-Ranked Canonical Pathway ^¥^	Hallmark Gene Sets/Network(s)© ^¥^	Function (Biological Process) ^¥^	Disease/Oncogenic Signature ^¥^	Top-Ranked Upstream Regulators
IPA	Antigen presentation pathway (1.38 × e^−9^)^®^ (0.282) ^€^ PD-L1 cancer immunotherapy pathway (4.68 × e^−8^) (0.132)	-	Connective tissue development and function, connective tissue disorders, organ morphology (25)	Nonpituitary endocrine tumour (5.07 × e^−8^)^®^ (240)	WDR5 (0.00906) NLRC5 (0.00906) TDP2 (0.0133)
GSEA	Androgen response (1.43 × e^−4^) (0.08) ^€^	AR pathway (0.0105) (0.082)	-	Cancer module 293 (1.61 × e^−7^), (0.5) (see Supplementary data S3)	-
GSEA (GO)	MHC protein complex (7.48 × e^−15^) (0.48)	Interferon gamma mediated signalling pathway (7.96 × e^−12^) (0.1667)	Antigen processing and presentation of peptide (1.53 × e^−10^) (0.1011)	-	-
GSEA (KEGG)	Allograft rejection (6.93 × e^−10^) (0.2703)	Pathways in cancer (3.14 × e^−5^) (14/328)	-	-	-
GSEA (REACTOME)	Interferon gamma signalling (8.75 × e^−12^) (0.1613)	MHC class II antigen presentation (2.33 × e^−6^) (0.0968)	-	-	-

© The hallmark gene sets represent the most-significant gene networks with the highest number of post-GWAS genes involved. ® FDR values for each pathway/gene set. € k/K ratio: k is the number of overlapped post-GWAS genes involved in the related pathways/gene sets and K is the number of total genes in the given pathway. IPA reports only k for the function and disease enrichment analysis. ¥ The value in the first and second parentheses represent FDR and k/K ratios, respectively.IPA: Ingenuity Pathway Analysis, GSEA: Gene Set Enrichment Analysis, KEGG: Kyoto Encyclopedia of Genes and Genomes, GO: Gene Ontology.

## Supplementary Materials

The following are available online at https://www.mdpi.com/2073-4425/11/5/526/s1. Table S1: Prostate cancer functional variants within coding/non-coding regions reported by post-GWAS. Table S2: Pathway analysis using the Ingenuity Pathway Analysis (IPA) algorithm. Table S3: Pathway and gene set analysis by GSEA, GO, KEGG and REACTOME. Table S4: Pathway analysis using the Ingenuity Pathway Analysis (IPA) algorithm for non-HLA genes. Table S5: Pathway and gene set analysis by GSEA, GO, KEGG and REACTOME for non-HLA genes. Table S6: The top-ranked/most significant canonical pathways, gene sets and molecular functions that non-HLA post-GWAS genes are enriched in. Table S7: The gene families that post-GWAS genes belong to, based on GSEA analysis. Figure S1: Genes regulated by CTNNB1 resulted from Ingenuity Pathway Analysis (IPA) upstream regulatory analysis.
