# Supplementary material for: Pathway Analysis of Genes Identified through Post-GWAS to Underpin Prostate Cancer Aetiology
Source: Genes (Basel). 2020 May 8;11(5):526. doi: 10.3390/genes11050526 (PMC7291227; doi:10.3390/genes11050526)
Supplement: Supplementary file 1 [file genes-11-00526-s001.zip › SupplementaryTable6.docx]

**Supplementary Table 6.** The top-ranked/most significant canonical pathways, gene sets and molecular functions that non-HLA post-GWAS genes are enriched in. In these analyses, we used the gene list described in Supplementary Table 1 excluding HLA genes.

| **Tool** | **Top-ranked canonical pathway**^¥^ | **Hallmark gene sets/ network(s)©**^¥^ | **Function (Biological Process)**^¥^ | **Disease/Oncogenic Signature**^¥^ | **Top-ranked upstream regulators** |  |  |
| --- | --- | --- | --- | --- | --- | --- | --- |
|  |  |  |  |  |  |  |  |
| **IPA** | Intrinsic Prothrombin Activation Pathway ( 0.0064)® ( 0.143)^€^ | - | Cell Morphology, Cellular Assembly and Organization, Organismal Injury and Abnormalities (27) | Nonpituitary endocrine tumor (1.82 e-7) (230) | WDR5 (0.00976)  TDP2 (0.0146)  EED (0.0146) |  |  |
| **GSEA** | AR pathway (0.0187) (0.082) | Androgen response (0.000104) (0.08) | - | Genes in the cancer module 3 (3.53 e-7) (0.0495), see :http://robotics.stanford.edu/~erans/cancer/modules/module_3 | - |  |  |
| **GO** | - | Cell cycle (1.36 e-6) (0.0244) | Embryo development (1.91 e-6) (0.0313) | *-* | - |  |  |
| **KEGG** | Pathways in cancer (0.00314) (0.04) | - | *-* | *-* | - |  |  |
| **REACTOME** | Abacavir transport and metabolism (0.00212) (0.4) | - | - | - | - |  |  |

**©**The hallmark gene sets represent most-significant gene networks with the highest number of post-GWAS genes involved.

®FDR values for each pathway/gene set.

**€** k/K ratio: k is the number of overlapped post-GWAS genes involved in the related pathways/gene sets and K is the number of total genes in the given pathway. IPA reports only k for the function and disease enrichments analysis.

¥ The value in the first and second parentheses represent FDR and k/K ratios, respectively.
