# Supplementary material for: Pathway Analysis of Genes Identified through Post-GWAS to Underpin Prostate Cancer Aetiology
Source: Genes (Basel). 2020 May 8;11(5):526. doi: 10.3390/genes11050526 (PMC7291227; doi:10.3390/genes11050526)
Supplement: Supplementary file 1 [file genes-11-00526-s001.zip › SupplementaryTable7.docx]

**Supplementary Table 7.** The gene families that post-GWAS genes belong to, based on GSEA analysis. In these analyses, we included HLA genes listed in Supplementary Table 1.2.

|  | [cytokines and growth factors](javascript:document.columnHeading0.submit();) | [transcription factors](javascript:document.columnHeading1.submit();) | [homeodomain proteins](javascript:document.columnHeading2.submit();) | [cell differentiation markers](javascript:document.columnHeading3.submit();) | [protein kinases](javascript:document.columnHeading4.submit();) | [translocated cancer genes](javascript:document.columnHeading5.submit();) | [oncogenes](javascript:document.columnHeading6.submit();) | [tumour suppressors](javascript:document.columnHeading7.submit();) |
| --- | --- | --- | --- | --- | --- | --- | --- | --- |
| [tumor suppressors](javascript:document.rowHeading0.submit();) | 0 | 0 | 0 | 0 | [1](javascript:document.intersection0_4.submit();) | 0 | 0 | [2](javascript:document.intersection0_7.submit();) |
| [oncogenes](javascript:document.rowHeading1.submit();) | 0 | [5](javascript:document.intersection1_1.submit();) | 0 | [1](javascript:document.intersection1_3.submit();) | [1](javascript:document.intersection1_4.submit();) | [9](javascript:document.intersection1_5.submit();) | [11](javascript:document.intersection1_6.submit();) |  |
| [translocated cancer genes](javascript:document.rowHeading2.submit();) | 0 | [5](javascript:document.intersection2_1.submit();) | 0 | 0 | 0 | [9](javascript:document.intersection2_5.submit();) |  |  |
| [protein kinases](javascript:document.rowHeading3.submit();) | 0 | 0 | 0 | [2](javascript:document.intersection3_3.submit();) | [8](javascript:document.intersection3_4.submit();) |  |  |  |
| [cell differentiation markers](javascript:document.rowHeading4.submit();) | 0 | 0 | 0 | [7](javascript:document.intersection4_3.submit();) |  |  |  |  |
| [homeodomain proteins](javascript:document.rowHeading5.submit();) | 0 | [9](javascript:document.intersection5_1.submit();) | [9](javascript:document.intersection5_2.submit();) |  |  |  |  |  |
| [transcription factors](javascript:document.rowHeading6.submit();) | 0 | [30](javascript:document.intersection6_1.submit();) |  |  |  |  |  |  |
| [cytokines and growth factors](javascript:document.rowHeading7.submit();) | [2](javascript:document.intersection7_0.submit();) |  |  |  |  |  |  |  |
