# Supplementary figures and images for: Pathway Analysis of Genes Identified through Post-GWAS to Underpin Prostate Cancer Aetiology
Source: Genes (Basel). 2020 May 8;11(5):526. doi: 10.3390/genes11050526 (PMC7291227; doi:10.3390/genes11050526)

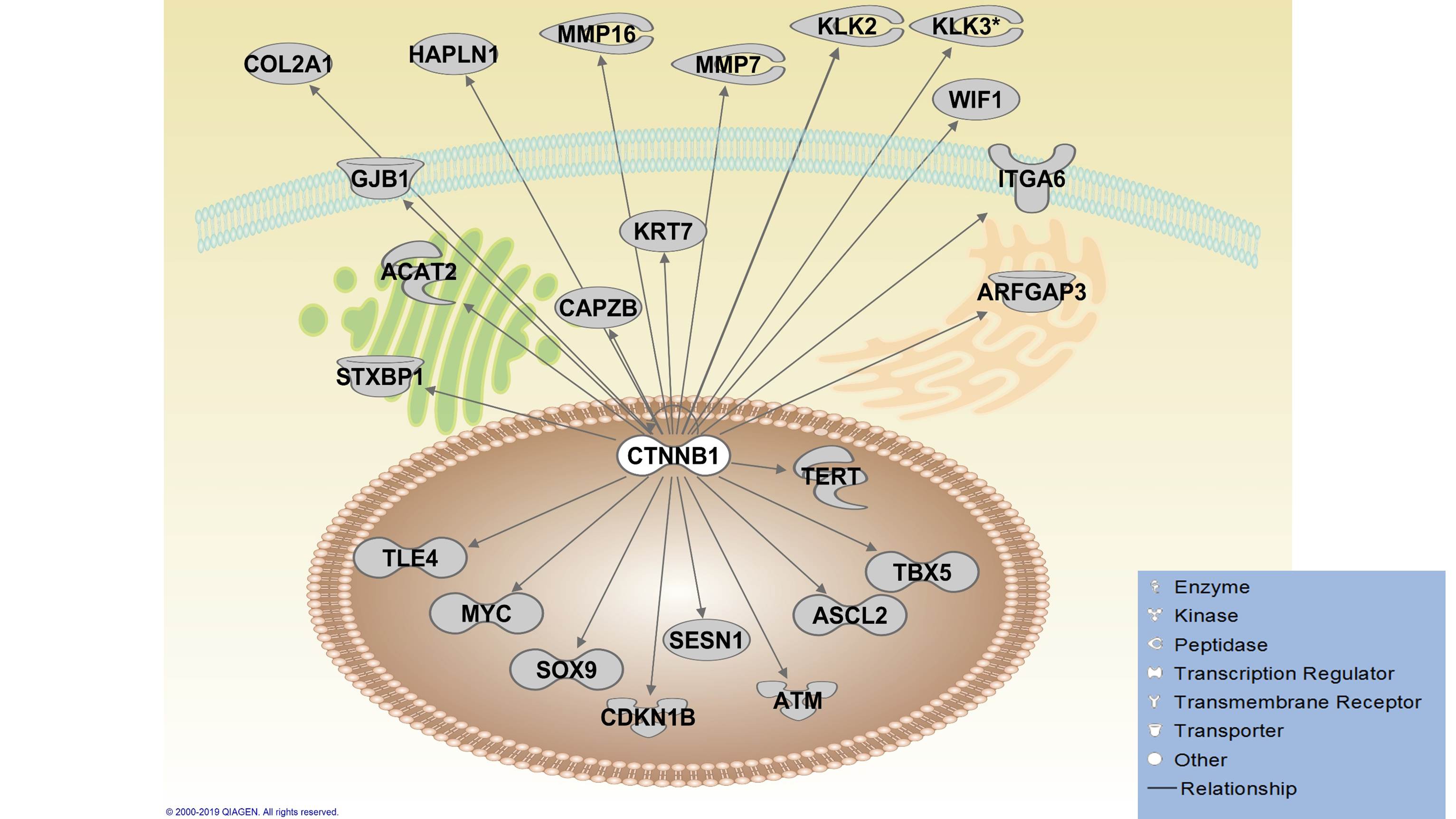

Supplement: Supplementary file 1 [file genes-11-00526-s001.zip › SupplementaryFigure1.jpg]
